# Evaluation of the Phytochemical Composition of Phenolic and Triterpene Compounds in Fruit of Large Cranberries (*Vaccinium macrocarpon* Aiton) Grown in Latvia

**DOI:** 10.3390/plants11202725

**Published:** 2022-10-15

**Authors:** Rima Šedbarė, Dace Siliņa, Valdimaras Janulis

**Affiliations:** 1 Department of Pharmacognosy, Faculty of Pharmacy, Lithuanian University of Health Sciences, 50166 Kaunas, Lithuania; 2 Faculty of Agriculture, Latvia University of Life Sciences and Technologies, 3001 Jelgava, Latvia

**Keywords:** cranberry, proanthocyanidins, flavonols, anthocyanins, triterpenoids

## Abstract

We carried out a qualitative and quantitative analysis of the phytochemical composition of the fruits of large cranberry cultivars ‘Ben Lear’, ‘Bergman’, ‘Kalnciema agra’, ‘Lemunyon’, ‘Pilgrim’, ‘Stevens’, and ‘Tina’ grown in Latvian climatic conditions. The following predominant compounds were found in cranberry fruit samples: peonidin-3-O-galactoside, peonidin-3-O-arabinoside, cyanidin-3-O-galactoside, cyanidin-3-O-arabinoside, myricetin-3-galactoside, quercetin-3-galactoside, quercetin-3-α-L-arabinofuranoside, quercetin 3-rhamnoside, ursolic acid, and oleanolic acid. During the berry ripening period (from 16 August until 15 September), a trend of decreasing amounts of compounds was found in the fruit samples of the studied cranberry cultivars: the total amount of proanthocyanidins decreased by 1.3 times, the total amount of the identified flavonols decreased by 1.3 times, the total amount of triterpenoids decreased by 1.2 times, and the total amount of chlorogenic acid decreased by 1.7 times. During the period from 16 August until 15 September, the total amount of anthocyanins in the cranberry fruit samples increased by 2.6 to 17 times. The highest total amount of anthocyanins (5305.80 ± 27 µg/g) was detected in fruit samples of the cranberry cultivar ‘Kalnciema agra’ collected on 15 September. The amount of biologically active compounds in cranberry fruit samples varies during berry ripening. Thus, the choice of the picking time is one of the factors that determines the phytochemical composition of raw cranberry material.

## 1. Introduction

Large cranberries (*Vaccinium macrocarpon* Ait.) are one of the widely cultivated berry crops in the food industry and are also used for the production of medicinal preparations and food supplements [1,2]. The majority (99%) of cranberries are grown in the USA, Canada, and Chile [3]. A small part of the global market production of cranberries is grown in Europe [3]. Scientific research is being carried out by introducing fruits, studying their phytochemical compositions, and searching for and identifying the most promising cultivars for growing in the Baltic region [1,4].

The climatic conditions of the Baltic region are favorable for the cultivation of cranberries [5]. This has led to the search for healthier nutritional options in modern society and encouraged the search for new cranberry cultivars and their cultivation as well as the creation of new food products and food supplements from raw cranberry fruit material [6,7]. During the development of cranberry cultivar selection and cultivation in the Baltic region, the main tasks of cultivation are to grow cranberry cultivars with known qualitative and quantitative compositions of biologically active compounds, whose fruits ripen early, are of uniform size and intense color, have a good yield, and are resistant to disease [8].

The biologically active compounds (proanthocyanidins, flavonols, anthocyanins, phenolic acids, and triterpene compounds) detected in large cranberry fruit determine their multifaceted pharmacological effects [9]. Peron et al. found that cranberry fruit preparations whose biologically active compounds are type A proanthocyanidins can be used for the treatment and prevention of urinary tract infections [10]. The systolic blood pressure and body mass index lowering effects of cranberry fruit preparations are associated with the antioxidant- and endothelium-dependent vasodilation-improving effects of quercetin [11]. The anthocyanins found in cranberry fruit suppress inflammatory processes in the intestines [12] and increase the amount and improve the composition of symbiotic intestinal bacteria [13]. The ursolic acid found in cranberry fruit has an anti-inflammatory effect [14] and inhibits the proliferation of liver and breast cancer cells [15].

Phytochemical composition studies are important for preparing high-quality raw plant material of the cultivated cranberry fruit, performing cranberry cultivar selection, and breeding new cultivars with more biologically active compounds in their fruits. The composition of secondary metabolites in cranberry fruit depends on the genetic characteristics of the plant and the effects of climatic conditions such as temperature, light, the chemical composition of the soil, and moisture [16]. Recently, methods in line with modern scientific progress have been applied (UPLC-DAD and UPLC-MS/MS) in the research of the phytochemical composition of cranberry fruits, which allows for an accurate evaluation of the changes in the qualitative and quantitative compositions of the biologically active compounds in cranberry fruit during the growing season.

The aim of our study was to investigate the phytochemical composition of anthocyanins, flavonols, chlorogenic acid, proanthocyanidins, and triterpenes in the large cranberry cultivars ‘Ben Lear’, ‘Bergman’, ‘Kalnciema agra’, ‘Lemunyon’, ‘Pilgrim’, ‘Stevens’, and ‘Tina’ grown in Latvia as well as the qualitative and quantitative changes of these compounds during berry ripening. The obtained results are important and provide new knowledge about changes in the phytochemical composition of cranberry fruit during berry ripening. These results can be used for further research in selecting suitable cranberry cultivars for their introduction and selection. 

## 2. Results and Discussions

### 2.1. Determination of the Qualitative and Quantitative Compositions of Anthocyanins

**Figure 1:**
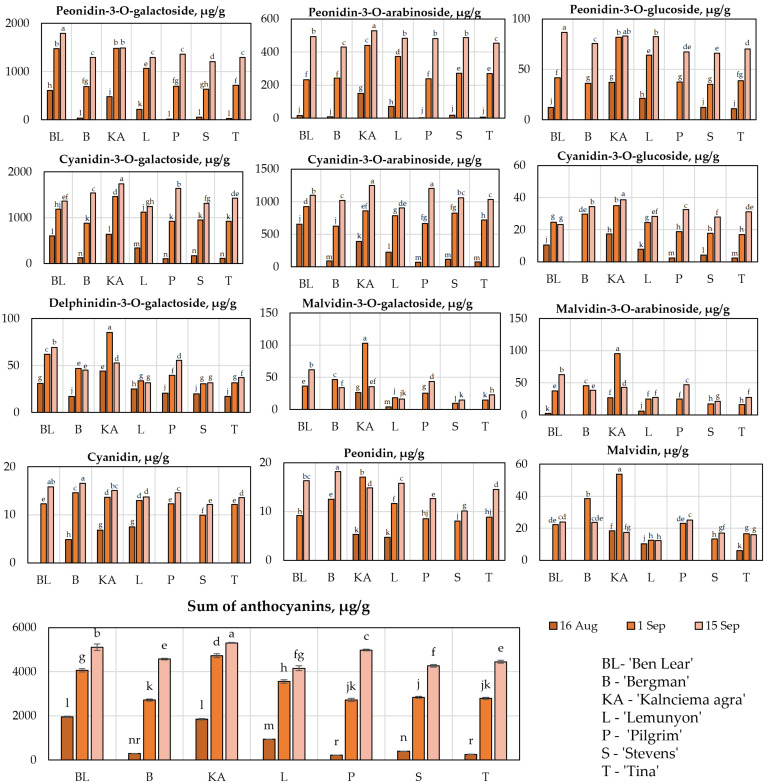
Variability in the qualitative and quantitative compositions of the anthocyanins and anthocyanidins in the fruit samples of the large cranberry cultivars. Different letters indicate statistically significant differences between the detected total anthocyanin contents in the cranberry fruit samples (*p* < 0.05).

Anthocyanins and anthocyanidins are a group of biologically active compounds found in cranberry fruit samples that determine their antioxidant and anti-inflammatory effects [17,18]. We conducted studies on the qualitative and quantitative composition of anthocyanins and anthocyanidins in the fruit of large cranberry cultivars ‘Ben Lear’, ‘Bergman’, ‘Kalnciema agra’, ‘Lemunyon’, ‘Pilgrim’, ‘Stevens’, and ‘Tina’ grown in Latvia (Figure 1).

The evaluation of the qualitative and quantitative compositions of anthocyanins and anthocyanidins showed that the samples of large cranberry cultivars contained four main glycoside compounds of the anthocyanin group: peonidin-3-O-galactoside, peonidin-3-O-arabinoside, cyanidin-3-O-galactoside, and cyanidin-3-O-arabinoside. The amount of peonidin-3-O-galactoside in the cranberry fruit samples during ripening varied from 6.77% to 36.24%, the amount of peonidin-3-O-arabinoside varied from 0.89% to 11.42%, the amount of cyanidin-3-O-galactoside varied from 26.66% to 50.32%, and the amount of cyanidin-3-O-arabinoside varied from 18.18% to 32.42%. Viškelis et al. studied fruit samples of cranberries grown in Lithuanian climatic conditions and found that the quantitative composition of the main anthocyanins was 32.7% peonidin-3-galactoside, 6.7% peonidin-3-arabinoside, 20.5% cyanidin-3-galactoside, and 19% cyanidin-3-arabinoside [6].

The evaluation showed that the total amount of anthocyanins and anthocyanidins in the cranberry fruit samples ranged from 222.79 ± 2 µg/g to 5305.80 ± 27 µg/g. The highest total amount of anthocyanins (5305.80 ± 27 µg/g) was found in fruit samples of the cranberry cultivar ‘Kalnciema agra’ collected on 15 September. The lowest total amount of anthocyanins (222.79 ± 2 µg/g) was found in fruit samples of the cranberry cultivar ‘Pilgrim’ collected on 16 August, which was not statistically significantly different from the amount detected in the fruit samples of the cranberry cultivar ‘Tina’ collected on 16 August (Figure 1).

Our study showed that from 16 August until 15 September the total amount of anthocyanins in the fruit samples of all the studied cultivars increased, with statistically significant differences between the samples of different studied cultivars. Our research showed that from 16 August to 1 September, the increase in the amount of anthocyanins was the most intense. In fruit samples of the cranberry cultivar ‘Kalnciema agra’, the total amount of anthocyanins increased by 2.5 times, and in fruit samples of the cranberry cultivar ‘Pilgrim’ the total amount of anthocyanins increased by 12 times. During the period from 1 September to 15 September, the total amount of anthocyanins in cranberry fruit increased, on average, by about 1.5 times in the samples of all the tested cranberry cultivars.

As cranberry fruit ripen, the amount of anthocyanins in cranberry fruit samples increases [19]. At the same time, changes in the morphological characteristics of the berries occur: berry size increases and the berry color changes from white to red [20]. Wang et al. found that in cranberry fruit anthocyanin biosynthesis begins at an early stage of fruit development, and the highest anthocyanin content is found in mature fruits, with significant differences between the tested cultivar samples [20].

Vvedenskaya et al. found in their study that the amount of anthocyanins in cranberry fruit samples of the ‘Ben Lear’ cultivar began to increase intensely from August 18, and in the cranberry fruit samples of the ‘Stevens’ cultivar, this increase occurred 3 weeks later on September 20 [21]. During their study, Wang et al. found that the amount of anthocyanins in fruit samples of the ‘Ben Lear’ cultivar started to increase on August 18, and in fruit samples of the ‘Stevens’ cultivar this increase started on August 28 [20]. Similar trends in the changes in the amounts of anthocyanins in fruit samples of ‘Ben Lear’ and ‘Stevens’ cultivars were also found during our study (Figure 1). Our study, as well as the studies of the aforementioned authors, showed that when comparing the amount of anthocyanins detected in fruit samples of the cranberry cultivars ‘Ben Lear’ and ‘Stevens’ collected at the same time, this amount was higher in the fruit samples of the ‘Ben Lear’ cultivar [20,21].

The fruit samples of the cranberry cultivar ‘Kalnciema Agra’ collected on 1 September differed from the samples of the other studied cultivars, as they had a higher content of malvidin, its glycosides, and delphinidin-3-O-galactoside. Fruit samples of the ‘Kalnciema agra’ cultivar contained 3 times more malvidin-3-O-galactoside, 2.2 times more malvidin-3-O-arabinoside, 3 times more malvidin, and 1.5 times more delphinidin-3-O-galactoside (Figure 1). The cranberry cultivar ‘Kalnciema agra’ was selected in 1998 by Alfreds Ripa in the National Botanical Garden. This cultivar is very early, and the berries ripen from the end of August to the beginning of September.

The amount of anthocyanins in cranberry fruit samples increased during berry ripening. Thus, the choice of the time for preparing cranberry fruit is one of the most important factors that determines the qualitative and quantitative compositions of anthocyanins in raw cranberry material. Considering the high total amounts of anthocyanins and malvidin group compounds found in fruit samples of the cranberry cultivar ‘Kalnciema agra’, this cultivar can be chosen for further introduction and the selection of cranberry cultivars.

### 2.2. Determination of Qualitative and Quantitative Compositions of Flavonols

**Figure 2:**
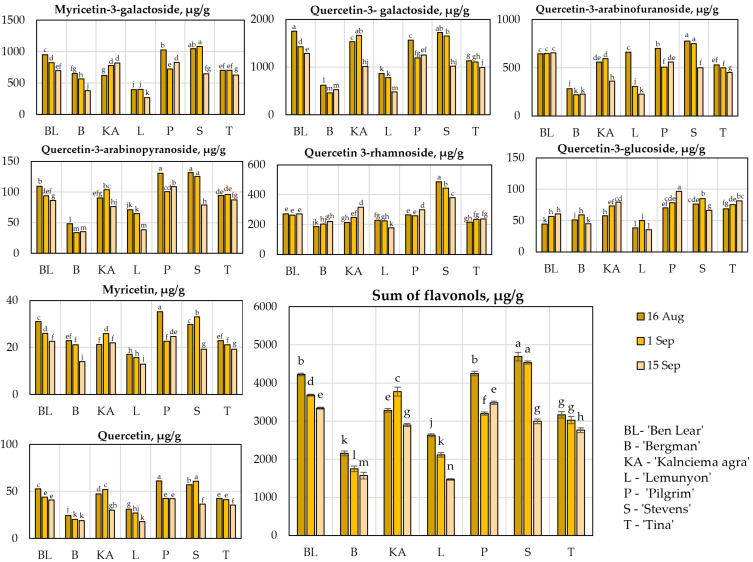
Variability in the qualitative and quantitative compositions of the flavonols in the fruit samples of the large cranberry cultivars. Different letters indicate statistically significant differences between the detected total amounts of flavonols in the cranberry fruit samples (*p* < 0.05).

Flavonols are a group of biologically active compounds whose antioxidant, cardioprotective, antibacterial, antiviral, and anticancer activities have been confirmed in both in vitro and in vivo studies [22,23,24]. A specific chromatographic profile of flavonol glycosides can be distinguished in the matrix of bioactive compounds of large cranberry fruits [25]. In our study, eight flavonol compounds were identified and quantified in cranberry fruit samples using the developed and validated UPLC-DAD methodology: myricetin-3-galactoside, quercetin-3-galactoside, quercetin-3-glucoside, quercetin-3-α-L-arabinopyranoside, quercetin-3-α-L-arabinofuranoside, quercetin 3-rhamnoside, myricetin, and quercetin (Figure 2).

The evaluation of the quantitative composition of flavanols showed that, in fruit samples of cranberry cultivars grown in Latvia, myricetin-3-galactoside, quercetin-3-galactoside, quercetin-3-α-L-arabinofuranoside, and quercetin 3-rhamnoside accounted for 91.16–93.85% of the total amount of the identified flavonols. In fruit samples, quercetin-3-galactoside accounted for 39.63% ± 4.36%, myricetin-3-galactoside accounted for 25.03% ± 4.36, quercetin-3-α-L-arabinofuranoside accounted for 17.80 ± 3.06%, and quercetin 3-rhamnoside accounted for 9.94% ± 2.52% of the total amount of flavonols. In their study, Wang et al. found that the quercetin-3-galactoside in the fruit samples of cranberry cultivars ‘Early Black’, ‘Howes’, ‘Ben Lear’, ‘Stevens’, ‘#35’, ‘Crimson Queen’, ‘Demoranville’, and ‘Mullica Queen’ accounted for 31–46%, myricetin-3-galactoside accounted for 19–32%, quercetin-3-α-L-arabinofuranoside accounted for 7–17%, and quercetin 3-rhamnoside accounted for 7–14% of the total amount of the identified flavonols [20].

The analysis of the quantitative composition showed that the total amount of flavonols in the fruit samples of the studied cranberry cultivars varied from 1256.45 ± 22 µg/g to 4327.29 ± 113 µg/g (Figure 2). The highest total amount of flavonols (4327. 29 ± 113 µg/g) was detected in the fruit samples cranberry of the ‘Stevens’ cultivar collected on 16 August, which was not statistically significantly different from the amount detected in fruit samples of the ‘Stevens’ cultivar collected on 1 September. The lowest total amount of the identified flavonols (1256.45 ± 22 µg/g) was detected in fruit samples of the ‘Lemunyon’ cultivar collected on 15 September.

During the ripening of cranberries (from 16 August until 15 September), the total amounts of the identified flavonols in the fruit samples of the studied cultivars ‘Ben Lear’, ‘Bergman’, ‘Kalnciema agra’, ‘Lemunyon’, ‘Pilgrim’, ‘Stevens’, and ‘Tina’ decreased by an average of 1.3 times (Figure 2). Wang et al. found that the levels of quercetin-3-galactoside and myricetin-3-galactoside in fruit samples of cultivars ‘Early Black’, ‘Howes’, ‘Ben Lear’, ‘Stevens’, ‘#35’, ‘Crimson Queen’, ‘Demoranville’, and ‘Mullica Queen’ changed only slightly during cranberry development and ripening [20]. Vvedenskaya et al. found that during the study period from 10 July until 11 October the total amount of flavonols in fruit samples of the ‘Ben Lear’ and ‘Stevens’ cultivars decreased by 1.3 and 1.4 times, respectively [21].

We found that the quantitative compositions of the flavonols in the cranberry samples changed only slightly during berry ripening. The results of our study correlate with those obtained by other researchers. Studies of the quantitative compositions of flavonols during berry ripening are important in the preparation of raw cranberry fruit material. They provide an opportunity to prepare a raw material with a known amount of flavonols, which can influence the biological effects of the preparations.

### 2.3. Determination of the Quantitative Composition of Proanthocyanidins and Chlorogenic Acid

The compounds of the proanthocyanidin group detected in cranberry fruits determine the biological effects of the preparations. A-type procyanidins suppress the adhesion of pathogenic bacterial strains to urinary tract epithelial cells [26]. Li et al. found that the consumption of cranberry juice with standardized proanthocyanidins inhibited Helicobacter pylori infection [27].

**Figure 3:**
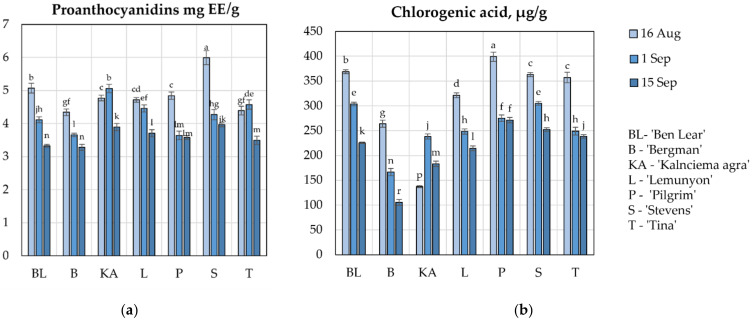
(**a**) Variability in the quantitative compositions of proanthocyanidins in the fruit samples of the large cranberry cultivars; (**b**) Variability in the quantitative composition of chlorogenic acid in the fruit samples of the large cranberry cultivars. Different letters indicate statistically significant differences between the detected amounts of the compounds in the cranberry fruit samples (*p* < 0.05).

Using the spectrophotometric method, we found that the total amounts of proanthocyanidins in the fruit samples of the studied cranberry cultivars ranged from 3.28 ± 0.11 mg EE/g to 5.99 ± 0.21 mg EE/g (Figure 3a). The highest amount of proanthocyanidins (5.99 ± 0.21 mg/g EE) was detected in the fruit samples of the ‘Stevens’ cultivar collected on 16 August, while the lowest amount (3.28 ± 0.11 mg EE/g) was detected in the fruit samples of the ‘Bergman’ cultivar collected on 15 September, which was not statistically significantly different from the amount detected in the fruit samples of the ‘Ben Lear’ cultivar collected on 15 September.

During the berry ripening period (from 16 August until 1 September), a trend of a 1.2-fold decrease in the amount of proanthocyanidins was found in the fruit samples of cranberry cultivars ‘Ben Lear’, ‘Bergman’, ‘Lemunyon’, ‘Pilgrim’, and ‘Stevens’. In fruit samples of the cultivars ‘Kalnciema agra’ and ‘Tina’ bred in Latvia, the concentrations of proanthocyanidins increased from 4.77 ± 0.09 mg/g to 5.06 ± 0.13 mg/g and from 4.39 ± 0.13 mg/g to 4.58 ± 0.14 mg/g, respectively (Figure 3a). During the ripening of cranberries (from 16 August until 15 September), the amounts of proanthocyanidins decreased by an average of 1.3 times in the fruit samples of all the studied cultivars, i.e., ‘Ben Lear’, ‘Bergman’, ‘Kalnciema agra’, ‘Lemunyon’, ‘Pilgrim’, ‘Stevens’, and ‘Tina’ (Figure 3a).

Vvedenskaya et al. and Wang et al. found that the concentration of proanthocyanidins decreased at the beginning of fruit ripening and slightly increased during the later stage of the ripening [20,21]. In their study, Vvedenskaya et al. found a higher amount of procyanidins in cranberry cultivar ‘Stevens’ compared to the amount found in fruit samples of the ‘Ben Lear’ cultivar during the berry ripening period [21]. We obtained similar results during our study.

Studies of the quantitative compositions of proanthocyanidins during berry ripening are important in the preparation of raw cranberry fruit materials. Research on the quantitative compositions of proanthocyanidins enables the preparation of high-quality raw material of cranberry fruits for the production of cranberry preparations and food supplements.

Chlorogenic acid has been found in the raw material of cranberry fruits. Galvez et al. indicated that chlorogenic acid reduces blood pressure, obesity, and dyslipidemia [28]. Oszmianski et al. indicated that the acids present in cranberry fruit, especially chlorogenic acid, are important because they determine the sensory properties of the berries and the products made from them [29].

The analysis of the quantitative composition of chlorogenic acid in the fruit samples of the cranberry cultivars grown in Latvia showed that the quantitative composition of chlorogenic acid varied from 105.54 ± 6 µg/g to 399.04 ± 9 µg/g (Figure 3b). The highest amount of chlorogenic acid (399.04 ± 9 µg/g) was detected in the fruit samples of the ‘Pilgrim’ cultivar collected on 16 August. The lowest amount of chlorogenic acid (105.54 ± 6 µg/g) was detected in fruit samples of the ‘Bergman’ cultivar collected on 15 September.

During the investigation period (from 16 August until 15 September), the amounts of chlorogenic acid in the fruit samples of the ‘Ben Lear’, ‘Bergman’, ‘Lemunyon’, ‘Pilgrim’, ‘Stevens’, and ‘Tina’ cultivars decreased by an average of 1.7 times (Figure 3b). The variation trends of chlorogenic acid content detected in the cranberry fruit samples of the ‘Kalnciema agra’ cultivar bred in Latvia differed from the trends of the variation of chlorogenic acid content in the other studied cultivars. The amount of chlorogenic acid detected in the cranberry fruit samples of the ‘Kalnciema agra’ cultivar increased by 1.7 times (1 September) and later (15 September) decreased by 1.3 times (Figure 3b).

The determination of the chlorogenic acid content in cranberry plant material can be applied for the assessment of quality and sensory properties of the berries. When preparing raw cranberry fruit material, the determination of the quantitative composition of chlorogenic acid enables a better prediction of the biological effects of the produced preparations.

### 2.4. Determination of the Qualitative and Quantitative Compositions of Triterpene Compounds

**Figure 4:**
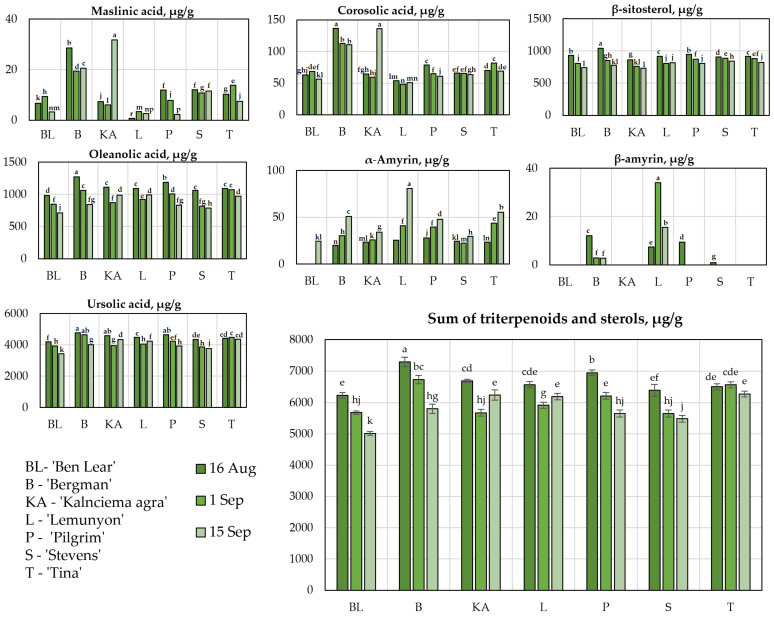
Variability in the qualitative and quantitative compositions of triterpene compounds in the fruit samples of the large cranberry cultivars. Different letters indicate statistically significant differences between the detected total amounts of the triterpene compounds in the cranberry fruit samples (*p* < 0.05).

Triterpene compounds are a group of biologically active compounds that have been shown to have dysmetabolism-preventing, anti-inflammatory, anticancer, and antioxidant effects [30,31]. Using our developed and validated UPLC-DAD methodology, triterpene compounds (maslinic acid, corosolic acid, oleanolic acid, ursolic acid, α-amyrin, and β-amyrin) and β-sitosterol (Figure 4) were identified and quantified in the cranberry fruit samples.

An analysis of the quantitative compositions of triterpene compounds was performed in the fruit samples of the large cranberry cultivars ‘Ben Lear’, ‘Bergman’, ‘Kalnciema agra’, ‘Lemunyon’, ‘Pilgrim’, ‘Stevens’, and ‘Tina’ grown in Latvia. We found that ursolic acid accounted for 68.31 ± 0.95% of the total quantitative composition of the triterpene compounds and phytosterols. In the fruit samples of the studied cranberry cultivars, the amounts of other triterpenoid compounds were significantly lower: the amount of oleanolic acid was 15.75 ± 0.95%, the amount of corosolic acid was 1.21 ± 0.37%, the amount of maslinic acid was 0.17 ± 0.13%, and the amount of α-amyrin was 0.52 ± 0.30%. 

The analysis of the quantitative composition of triterpenes revealed that the amounts of triterpenoids in the cranberry fruit samples ranged from 4215.78 ± 45 µg/g to 6232.74 ± 123 µg/g (Figure 4). The highest total amount of triterpene compounds (6232.74 ± 123 µg/g) was detected in the fruit samples of the ‘Bergman’ cultivar collected on 16 August, while the lowest total amount of the identified triterpenoids (4215.78 ± 45 µg/g) was detected in the fruit samples of the ‘Ben Lear’ cultivar collected on 15 September (Figure 4).

The contents of β-sitosterol in the fruit samples of cranberry cultivars ranged from 733.88 ± 21 µg/g to 1040.98 ± 33 µg/g. The highest β-sitosterol content (1040.98± 33 µg/g) was detected in the fruit samples of the ‘Bergman’ cultivar collected on 16 August, while the lowest β-sitosterol content (733.88 ± 21 µg/g) was found in the fruit samples of the ‘Kalnciema agra’ cultivar collected on 15 September, which was not statistically significantly different from the amount detected in the fruit samples of the cranberry cultivar ‘Ben Lear’ collected on 15 September.

During the study period (from 16 August until 15 September), the contents of ursolic acid, oleanolic acid, and β-sitosterol in the cranberry fruit samples decreased by about 1.2 times. Oszmianski et al. found that during the ripening period the concentrations of triterpenoids (ursolic acid, oleanolic acid, and betulinic acid) in cranberry fruit samples of the ‘Pilgrim’, ‘Stevens’, and ‘Ben Lear’ cultivars increased by an average of 1.2 times from the beginning of berry ripening to the fully ripe stage of the berries [32].

The quantitative compositions of corosolic acid, maslinic acid, β-amyrin, and α-amyrin in the cranberry fruit samples of the tested cultivars varied during ripening. The highest amount of maslinic acid (31.80 ± 1 µg/g) was found in the ‘Kalnciema agra’ samples collected on 15 September, and the highest amount of corosolic acid (136.26 ± 2 µg/g) was found in ‘Kalnciema agra’ samples collected on 15 September, which was not statistically significantly different from the amount detected in the fruit samples of the ‘Bergman’ cultivar collected on 16 August. In the fruit samples of all studied cranberry cultivars, during the ripening period (from 16 August until 15 September), the amount of α-amyrin varied. In the fruit samples of the ‘Ben Lear’ cultivar, it increased to 24.42 ± 2 µg/g, in the ‘Bergman’ cultivar it increased from 19.99 ± 1.5 µg/g to 51.02 ± 0.8 µg/g, in the ‘Kalnciema agra’ cultivar it increased from 23.52 ± 2 µg/g to 34.18 ± 2 µg/g, in the ‘Lemunyon’ cultivar it increased from 25.62 ± 3 µg/g to 80.79 ± 4 µg/g, in the ‘Pilgrim’ cultivar it increased from 28.00 ± 1 µg/g to 47.67 ± 2 µg/g, in the ‘Stevens’ cultivar it increased from 24.56 ± 2 µg/g to 29.77 ± 3 µg/g, and in the fruit samples if the ‘Tina’ cultivar it increased from 23.54 ± 1.6 µg/g to 55.52 ± 3 µg/g. β-amyrin was detected in the fruit samples of the ‘Lemunyon’, ‘Bergman’, ‘Pilgrim’, and ‘Stevens’ cranberry cultivars. The highest β-amyrin content (34.03 ± 3 µg/g) was detected in the fruit samples of the cranberry cultivar ‘Lemunyon’ collected on 1 September. During the study period (from 16 August until 15 September), the amounts of this compound in the cranberry fruit samples of the ‘Lemunyon’ and ‘Bergman’ cultivars were found to be, respectively, two and four times lower. β-amyrin was not detected in the fruit samples of the ‘Pilgrim’ or ‘Stevens’ cultivars collected on 15 September (Figure 4).

Studies of the phytochemical compositions of cranberry plant raw materials enable the identification of triterpene compounds, their quantitative compositions, and trends in their variability. Cranberries are a botanical source of ursolic acid. Thus, cranberry plant material could be used in the development and production of preparations with ursolic acid. 

### 2.5. Principle Component Analysis of the Distributions of Compounds Identified in Samples of Cranberry Cultivars during Berry Ripening

**Figure 5:**
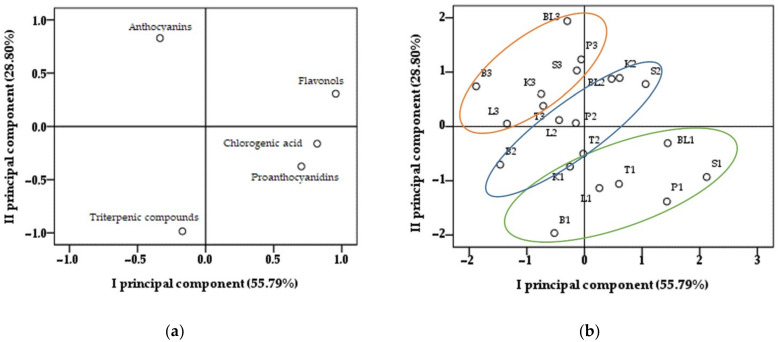
Principal component analysis loading (**a**) and score (**b**) plots of different cranberry samples. (**b**) The samples of cultivars collected on 16 August: 1BL—’Ben Lear’, 1B—‘Bergman’, 1KA—’Kalnciema agra’, 1L—‘Lemunyon’, 1P—‘Pilgrim’, 1S—‘Stevens’, and 1T—‘Tina’; the samples of cultivars collected on 1 September: 2BL—‘Ben Lear’, 2B—‘Bergman’, 2KA—‘Kalnciema agra’, 2L—‘Lemunyon’, 2P—‘Pilgrim’, 2Sf—‘Stevens’, and 2T—‘Tina’; the samples of cultivars collected on 15 September: 3BL—‘Ben Lear’, 3B—‘Bergman’, 3KA—‘Kalnciema agra’, 3L—‘Lemunyon’, 3P—‘Pilgrim’, 3S—‘Stevens’, and 3T—‘Tina’.

In order to evaluate the quantitative variability in anthocyanins, flavonols, proanthocyanidins, chlorogenic acid, and triterpene compounds found in the samples of large cranberry cultivars during the ripening of cranberry fruit, a principal component analysis was performed (Figure 5). Two principal components were used for the analysis, explaining 84.59% of the total variance in the data. Principal component I (I PC), which describes 55.79% of the total data variance, correlates with the following compounds: flavonols (0.890), chlorogenic acid (0.854), proanthocyanidins (0.784), and anthocyanidins (−0.514) (Figure 5a). Principal component II (II PC), which describes 28.80% of the total data variance, correlates with the following compounds: proanthocyanidins (−0.527), triterpenic compounds (−0.948), and anthocyanidins (0.901) (Figure 5a). 

The fruit samples of large cranberries collected on 16 August were located in the negative II PC area (Figure 5b). During this fruit collection, cranberry fruit samples were found to contain the highest amounts of proanthocyanidins, flavonols, chlorogenic acid, and triterpene lignans, while the amount of anthocyanins was small. Vvedenskaya et al. found that the highest amounts of proanthocyanidins and flavonols were detected in the early stage of cranberry fruit development and claimed that flavonoids have a protective effect against pathogenic fungi [21].

The arrangement of the cranberry fruit samples collected on 1 September was shifted to the center of the chart of the principal component analysis (Figure 5b). During the study, we found that the amounts of anthocyanins and anthocyanidins in the cranberry fruit samples increased by an average of seven times as the berries ripened. Meanwhile, the amounts of proanthocyanidins, chlorogenic acid, flavonols, and triterpene compounds decreased. The fruit samples of the cranberry cultivars ‘Ben Lear’, ‘Kalnciema agra’, and ‘Stevens’ collected on 1 September were located in the positive I PC and positive II PC area due to the higher contents of flavonols found during the study.

The arrangement of fruit samples of the cranberry cultivars collected on 15 September shifted to the negative I PC and positive II PC area (Figure 5b). The amounts of anthocyanins and anthocyanidins in the fruit samples of cranberry cultivars collected on 15 September increased by an average of 1.5 times, while the amounts of proanthocyanidins, chlorogenic acid, flavonols, and triterpene compounds decreased.

Forney et al. found that during the ripening of cranberry fruit the changes in the total amounts of phenolic compounds were not large, but changes in the compositions of individual phenolic compounds may have a greater significance for the nutritional value of cranberries [33]. During berry ripening, the quantitative compositions of anthocyanins and anthocyanidins in the cranberry fruit samples changed the most. Karppinen et al. stated that during berry ripening there was an increase in the transcription levels of the enzymes that determine anthocyanin accumulation, namely chalcone synthase (CHS), dihydroflavonol 4-reductase (DFR), anthocyanidin synthase (ANS), and UDP-glucose flavonoid 3-O-glucosyltransferase (UFGT) [16]. The effect of light on fruits is one of the factors that determines the rate of the ripening of berries [34]. Zhou et al. found that the synthesis of individual anthocyanins in cranberry fruit is catalyzed differently by natural light, red light, and far-red light. Under the influence of natural light, the total amount of anthocyanins in cranberry fruits increased by 75.3% in 24 h and by 87.2% within 48 h compared to the control sample kept in the dark [35].

As cranberry fruits ripen, the composition of the biologically active compounds changes. Thus, determining the phytochemical composition of raw cranberry plant material is important in order to provide the consumer with herbal preparations of high commercial value and known compositions. The highest amounts of anthocyanins in the cranberry fruit samples were detected during the third picking, and the highest amounts of triterpene compounds, flavonols, proanthocyanidins, and chlorogenic acid were detected at the early stage of berry ripening. The selection of the time of harvesting raw material for cranberry fruit is one of the important factors that determines the compositions of biologically active compounds in the berries. By applying the principal component analysis, we found that raw cranberry material prepared at the beginning of September will have a higher content of proanthocyanidins, flavonols, and chlorogenic acid. Raw cranberry material prepared in the second half of September will have a large amount of anthocyanins. Routine studies of the qualitative and quantitative compositions of the biologically active compounds of cranberry plant material would allow for better control of the compositions of cranberry plant material and the preparations made from it and for the prediction of specific pharmacological effects [36,37].

## 3. Materials and Methods

### 3.1. Reagents

The following reagents were used in the study: acetone, methanol (Sigma-Aldrich, Steinheim, Germany), ethanol 96% (*v*/*v*) (AB Stumbras, Kaunas, Lithuania), formic acid (Merck, Darmstadt, Germany), acetonitrile (Sigma-Aldrich, Steinheim, Germany), hydrochloric acid (Sigma-Aldrich, Steinheim, Germany), and 4-(Dimethylamino)cinnamaldehyde (Sigma-Aldrich, Steinheim, Germany). Reference standards for maslinic acid, corosolic acid, oleanolic acid, ursolic acid, β-amyrin, α-amyrin, β-sitosterol, chlorogenic acid, myricetin, quercetin-3-rhamnoside, quercetin-3-α-L-arabinofuranoside, and quercetin-3-α-L-arabinopyranoside were obtained from Sigma-Aldrich (Steinheim, Germany). Reference standards for delphinidin-3-galactoside, cyaniding-3-galactoside, cyaniding-3-glucoside, cyaniding-3-arabinoside, peonidin-3-galactoside, peonidin-3-arabinoside, peonidin-3-glucoside, malvidin-3-galactoside, malvidin-3-arabinoside, cyanidin chloride, peonidin chloride, malvidin chloride, and myricetin-3-galactoside were purchased from Extrasynthese (Genay, France). Reference standards for quercetin-3-galactoside and quercetin were obtained from Carl Roth (Karlsruhe, Germany). The reference standard for quercetin-3-O-glucoside was obtained from Biochemistry (Buchs, Switzerland). The purified deionized water used in the experiments was prepared using a Milli-Q^®^ (Millipore, Bedford, MA, USA) water purification system.

### 3.2. Raw Material

The object of the study was large cranberry (*Vaccinium macrocarpon* Aiton.) samples of different cultivars grown in Latvian climatic conditions, which were obtained from the collection of the Latvia University of Life Sciences and Technologies. Latvia has a midlatitude climate, transitioning from maritime to continental [38]. The average annual temperature is 6.8 °C [39]. Samples of the cultivars ‘Lemunyon’ and ‘Bergman’ were obtained from the collection grown on Strazdu Street 1 in Jelgava (56°39′47.1″ N, 23°45′13.2″ E). Samples of the cultivars ‘Ben Lear’, ‘Kalnciema Agra’, ‘Pilgrim’, ‘Stevens’, and ‘Tina’ were collected from the large cranberry plantation grown on “Vilku purvs” (56°35′06.0″ N, 24°35′37.0″ E). The samples were collected three times: on 16 August, 1 September, and 15 September 2021. Cranberry fruits were frozen at −20 °C in a freezer then at −60 °C in an ultra-low-temperature freezer (CVF330/86, ClimasLab SL, Barcelona, Spain). Cranberry fruits were freeze-dried according to the methodology described by Gudžinskaitė et al. [40]. The fruits were powdered in a Retsch GM 200 electric mill (Retsh GmbH, Hahn, Germany). Loss on drying was determined using the method described in the European Pharmacopoeia Ph.Eur.01/2008: 2023 [41].

### 3.3. Preparation of Cranberry Extracts

The extraction of anthocyanins and phenolic compounds from cranberry fruit samples was performed according to the methodology described by Urbstaite et al. [42,43]. The extraction of triterpenic compounds and phytosterol compounds from cranberry fruit samples was performed according to the methodology described by Sedbare et al. [44]. Prior to the chromatographic analysis, the cranberry extracts were filtered through membrane filters with 0.22 µm pores (Carl Roth GmbH, Karlsruhe, Germany).

### 3.4. Chromatographic Analysis

The analysis of the qualitative and quantitative compositions of the anthocyanins, phenolic compounds, and triterpenic compounds in cranberry fruit was performed using a Waters ACQUITY Ultra High-Performance LC system (Water, Milford, MA, USA) with a photodiode array detector. An ACE C18 reversed-phase column (ACT, Aberdeen, UK; 100 × 2.1 mm, 1.7 µm particle size) was used for the separation of the compounds. 

The analysis of the qualitative and quantitative compositions of the anthocyanins in the cranberry fruit was performed using the ultra-high-performance liquid chromatography methodology validated by Vilkickyte et al. [45]. Gradient separation was achieved using solvent A (a 10% formic acid aqueous solution) and solvent B (acetonitrile) at a flow rate of 0.5 mL/min and the following gradient change: 0.0–2.0 min, 95% A; 2.0–7.0 min, 91% A; 7.0–9.0 min, 88% A; 9.0–10.0 min, 75% A; 10.0–10.5 min, 20% A; 10.5–11.0 min, 20% A; and 11.0–12.0 min, 95% A. A chromatogram of the identified peaks of the cranberry fruit sample and their linearity parameter are included in the Supplementary Materials (Figure S1 and Table S1).

The determination of the phenolic compounds in the cranberry fruit extracts was performed according to the methodology developed and validated by Urbstaite et al. [42]. Gradient separation was achieved using solvent A (a 0.1% formic acid aqueous solution) and solvent B (acetonitrile) at a flow rate of 0.5 mL/min and the following gradient change: 0 min, 95% A; 1 min, 88% A; 3 min, 88% A; 4 min, 87% A; 9 min, 75% A; 10.5 min, 70% A; 12 min, 70% A; 12.5 min, 10% A; 13 min, 10% A; 13.5 min, 95% A; and 14.5 min, 95% A, delaying the next injection by 2 min. A chromatogram of the identified peaks of the cranberry fruit sample and their linearity parameter are included in the Supplementary Materials (Figure S2 and Table S2).

The analysis of the compositions of the triterpene compounds in the cranberry fruits was performed using the methodology developed and validated by Sedbare et al. [44]. Gradient separation was achieved using solvent A (a 0.1% formic acid aqueous solution) and solvent B (methanol) at a flow rate of 0.2 mL/min and the following gradient change: 0 min, 8% A; 8 min, 3% A; 9 min, 2% A; 29.5 min, 2% A; and 30 min, 8% A, delaying the subsequent injection by 10 min. A Chromatogram of the identified peaks of the cranberry fruit sample and their linearity parameter are included in the Supplementary Materials (Figure S3 and Table S3).

A compound identification analysis was performed by comparing the UV absorption spectrum of the reference standard with the UV absorption spectrum of the large cranberry matrix peaks using the same retention time and based on literature data [20,46,47,48].

### 3.5. Spectrophotometric Studies

The total content of proanthocyanidins was determined by the DMCA (4-(Dimethylamino)cinnamaldehyde) assay [49]. First, 10 µL of cranberry extract was mixed with 3 mL of a 0.1% DMCA reagent solution in acidified ethanol (9:1, *v*/*v*). The reference solution was a DMCA solution in acidified ethanol. After 5 min, the absorption was measured with an M550 UV/Vis spectrophotometer (Spectronic CamSpec, Garforth, UK) at λ = 640 nm. The total content of proanthocyanidins was calculated from the (–)-epicatechin (0.0625 mg/mL–1 mg/mL) calibration curve (*y* = 0.7021*x* + 0.0138; R^2^ = 0.9994) and is expressed as mg/g (–)-epicatechin equivalent (EE) dry weight.

### 3.6. Statistical Analysis

Data analysis was performed using the computer software programs Microsoft Excel 2016 (Microsoft, Redmond, WA, USA) and SPSS Statistics 21 (IBM, Armonk, NY, USA). During the study, the means and standard deviations (SD) of the three independent evaluations were calculated. To evaluate the differences in the amounts of anthocyanins, anthocyanidins, flavonols, chlorogenic acid, proanthocyanidins, and triterpenic compounds between samples of cranberry cultivars, a one-way analysis of variance with Tukey’s test for multiple comparisons was used. The quantitative analysis data were further analyzed by applying a principal component analysis (PCA). Differences at *p* < 0.05 were statistically significant.

## 4. Conclusions

The choice of the time of the collection of cranberry fruit samples is one of the important factors that determines the phytochemical composition of raw cranberry material. By applying the principal component analysis, we found that raw cranberry material prepared at the beginning of September will have higher contents of proanthocyanidins, flavonols, chlorogenic acid, triterpenoids, and β-sitosterol. Raw cranberry material prepared in the second half of September will have large amounts of anthocyanins and anthocyanidins. Studies of the qualitative and quantitative compositions of the biologically active compounds of cranberry fruit samples provide an opportunity to prepare raw material with a known amounts of biologically active compounds that may influence the biological effects of the preparations.

## Data Availability

All data generated during this study are included in this article.

## Supplementary Materials

The following supporting information can be downloaded at: https://www.mdpi.com/article/10.3390/plants11202725/s1, Figure S1: UHPLC-PDA chromatogram (λ = 520 nm) of the large cranberry extract (A); anthocyanins and anthocyanidins standard mix (B). The compounds of the identified peaks are described in Table S1 (linearity parameters of the identified anthocyanins and anthocyanidins); Figure S2: UHPLC-PDA chromatogram (λ = 360 nm) of the large cranberry extract (A); chlorogenic acid and flavonols standard mix (B). The compounds of the identified peaks are described in Table S2 (linearity parameters of the identified chlorogenic acid and flavonols); Figure S3: UHPLC-PDA chromatogram (λ = 205.5 nm) of the large cranberry extract (A); β-sitosterol and triterpenoids standard mix (B). The compounds of the identified peaks are described in Table S3 (linearity parameters of the identified β-sitosterol and triterpenoids).
