# Supplementary material for: Evaluation of the Phytochemical Composition of Phenolic and Triterpene Compounds in Fruit of Large Cranberries (Vaccinium macrocarpon Aiton) Grown in Latvia
Source: Plants (Basel). 2022 Oct 15;11(20):2725. doi: 10.3390/plants11202725 (PMC9609109; doi:10.3390/plants11202725)
Supplement: Supplementary file 1 [file plants-11-02725-s001.zip › plants-1982052-supplementary.pdf]

Supplementary material:

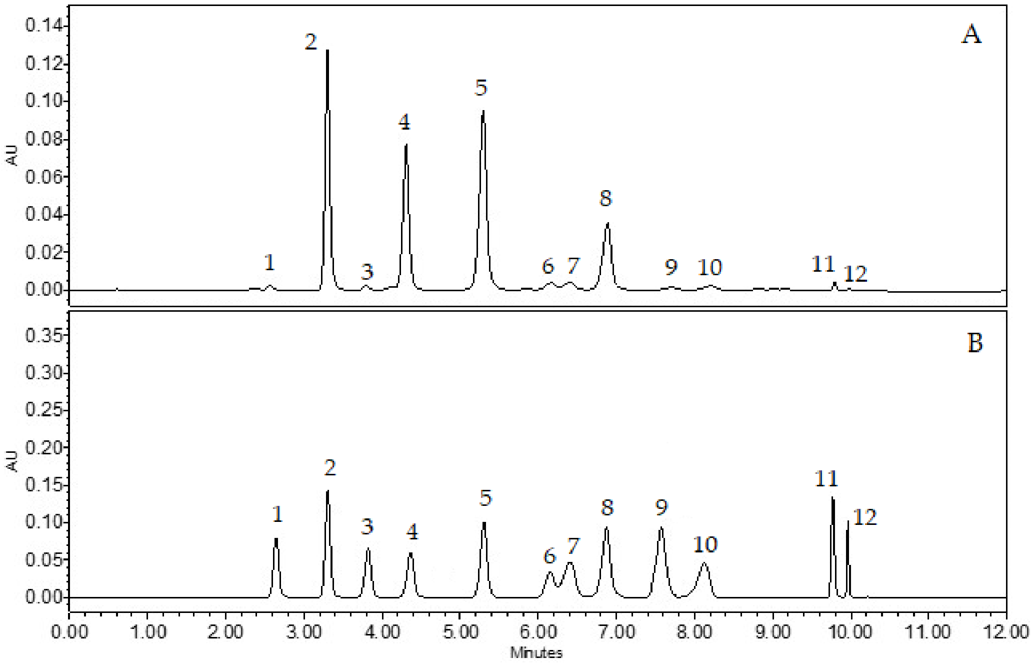

Figure S1: UHPLC-PDA chromatogram (λ = 520 nm) of the large cranberry extract (A); anthocyanins and anthocyanidins standart mix (B). The compounds of the identified peaks are described in Table S1.

Table S1. Linearity parameters of the identified anthocyanins and anthocyanidins.

Peak	Compound	Calibration equation	Linearity range (μg/mL)	R^2
1	Delphinidin-3-galactoside	$y = 4900\ x - 3480$	3.91–125.00	0.999
2	Cyanidin-3-galactoside	$y = 4940\ x - 2370$	0.78–125.00	0.999
3	Cyanidin-3-glucoside	$y = 4230\ x + 1610$	3.13–100.00	0.999
4	Cyanidin-3-arabinoside	$y = 4800\ x + 2220$	3.13–100.00	0.999
5	Peonidin-3-galactoside	$y = 5320\ x + 8770$	3.125–100	0.999
6	Peonidin-3-glucoside	$y = 3970\ x - 1430$	0.98–125.00	0.999
7	Malvidin-3-galactoside	$y = 6890\ x + 3110$	3.13–100.00	0.999
8	Peonidin-3-arabinoside	$y = 5940\ x + 5320$	3.125–100	0.999
9	Cyanidin	$y = 10400\ x - 1930$	0.78–100.00	0.999
10	Malvidin-3-arabinoside	$y = 5950\ x + 1590$	0.78–125.00	0.999
11	Peonidin	$y = 7010\ x + 1020$	1.56–100.00	0.999
12	Malvidin	$y = 1150\ x + 171$	3.13–100.00	0.999

Table S2. Linearity parameters of the identified chlorogenic acid and flavonols.

Peak	Compound	Calibration equation	Linearity range (μg/mL)	R^2
1	Chlorogenic acid	$y = 5060\ x + 570$	1.95–62.5	0.999
2	Myricetin-3-galactoside	$y = 3450\ x - 396$	0.78–100	0.999
3	Quercetin-3-galactoside	$y = 4880\ x + 1180$	3.13–200	0.999
4	Quercetin-3-glucoside	$y = 4160\ x - 61{,}7$	3.13–50	0.999
5	Quercetin-3-α-L-arabinopyranoside	$y = 5250\ x + 861$	3.13–50	0.999
6	Quercetin-3-α-L-arabinofuranoside	$y = 4170\ x - 199$	3.13–50	0.999
7	Quercetin-3-rhamnoside	$y = 3690\ x + 797$	3.13–50	0.999
8	Myricetin	$y = 5360\ x - 1240$	1.56–50	0.999
9	Quercetin	$y = 7450\ x - 1070$	3.13–50	0.999

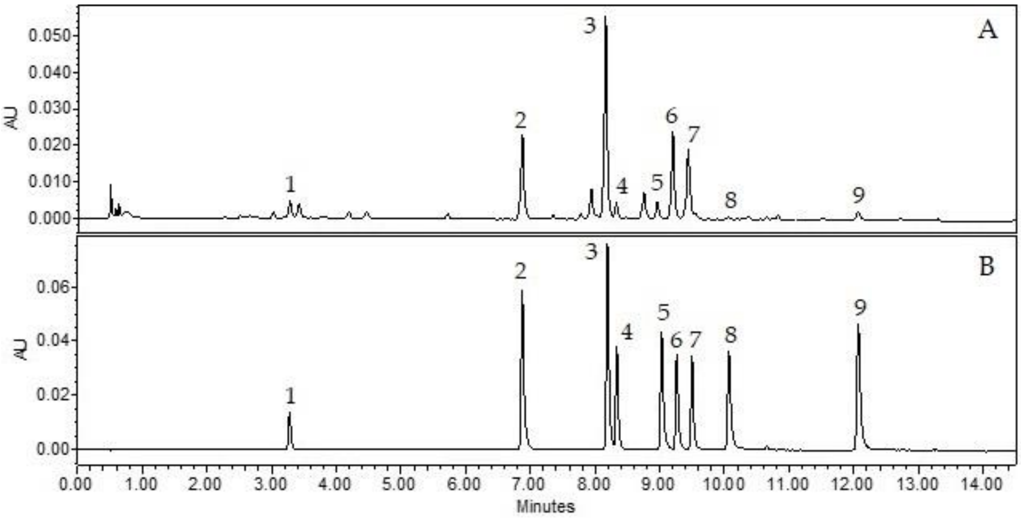

Figure S2: UHPLC-PDA chromatogram (λ = 360 nm) of the large cranberry extract (A); chlorogenic acid and flavonols standard mix (B). The compounds of the identified peaks are described in Table S2.

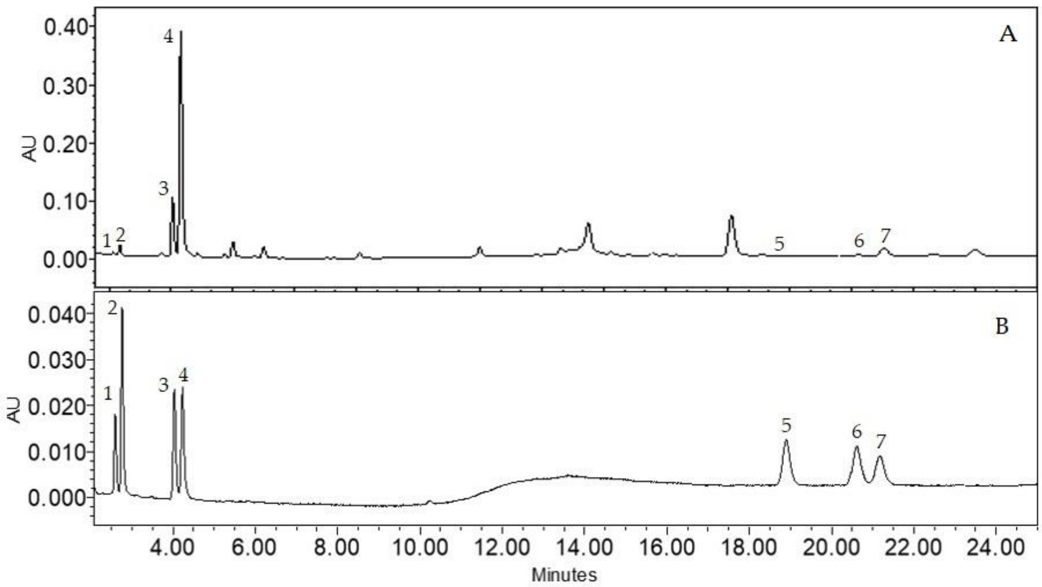

Figure S3: UHPLC-PDA chromatogram (λ = 205.5 nm) of the large cranberry extract (A); β-Sitosterol and triterpenoids standard mix (B). The compounds of the identified peaks are described in Table S3.

Table S3. Linearity parameters of the identified β-Sitosterol and triterpenoids.

Peak	Compound	Calibration equation	Linearity range (μg/mL)	R^2
1	Maslinic acid	$y = 2790\ x + 3990$	3.125–200	0.999
2	Corosolic acid	$y = 3280\ x + 750$	3.125–200	0.999
3	Oleanolic acid	$y = 3240\ x + 12900$	2.344–600	0.999
4	Ursolic acid	$y = 2930\ x + 39000$	3.906–2000	0.999
5	β-Amyrin	$y = 3170\ x + 6470$	6.250–200	0.999
6	α-Amyrin	$y = 3090\ x – 1030$	6.250–200	0.999
7	β-Sitosterol	$y = 2100\ x + 4830$	6.250–200	0.999
